# Mesenchymal Stem Cells (MSC) Regulate Activation of Granulocyte-Like Myeloid Derived Suppressor Cells (G-MDSC) in Chronic Myeloid Leukemia Patients

**DOI:** 10.1371/journal.pone.0158392

**Published:** 2016-07-08

**Authors:** Cesarina Giallongo, Alessandra Romano, Nunziatina Laura Parrinello, Piera La Cava, Maria Violetta Brundo, Vincenzo Bramanti, Fabio Stagno, Paolo Vigneri, Annalisa Chiarenza, Giuseppe Alberto Palumbo, Daniele Tibullo, Francesco Di Raimondo

**Affiliations:** 1 Division of Hematology, A.O.U. Policlinico-OVE, Catania, University of Catania, Catania, Italy; 2 Department of Biomedical and Biotechnological Sciences, University of Catania, Catania, Italy; 3 Department of Biological, Geological, and Environmental Sciences, University of Catania, Catania, Italy; 4 Oncology, Department of Clinical and Experimental Medicine, University of Catania, Catania, Italy; University of Texas M.D. Anderson Cancer Center, UNITED STATES

## Abstract

It is well known that mesenchymal stem cells (MSC) have a role in promotion of tumor growth, survival and drug-resistance in chronic myeloid leukemia (CML). Recent reports indicated that a subpopulation of myeloid cells, defined as granulocyte-like myeloid-derived suppressor cells (G-MDSC) is increased in these patients. So far, the role of MSC in MDSC expansion and activation into the BM microenvironment remains unexplored. To address this question, here we use a specific experimental model in vitro, co-culturing MSC with peripheral blood mononucleated cells (PBMC) from normal individuals, in order to generate MSC-educated G-MDSC. Although MSC of healthy donors (HD) and CML patients were able to generate the same amount of MDSC, only CML-MSC-educated G-MDSC exhibited suppressive ability on autologous T lymphocytes. In addition, compared with HD-MSC, CML-MSC over-expressed some immunomodulatory factors including TGFβ, IL6 and IL10, that could be involved in MDSC activation. CML-MSC-educated G-MDSC expressed higher levels of ARG1, TNFα, IL1β, COX2 and IL6 than G-MDSC isolated from co-culture with HD-MSC. Our data provide evidence that CML-MSC may play a critical role in tumor microenvironment by orchestrating G-MDSC activation and regulating T lymphocytes-mediated leukemia surveillance, thus contributing to CML immune escape.

## Introduction

Chronic myeloid leukemia (CML) is a hematopoietic stem cell malignancy characterized by the t(9;22) chromosomal translocation that generates the BCR/ABL oncogene [1]. BCR-ABL tyrosine kinase inhibitors (TKI) are able to induce remission in CML patients but not to eliminate leukemia stem cells (LSC), which can regenerate leukemia on drug discontinuation [2–4]. Understanding LSC regulation is critical to understand CML pathogenesis and to develop curative strategies. Proliferation, survival and drug-resistance of leukemic cells are largely dependent on their interplay with the bone marrow (BM) microenvironment, in which mesenchymal stem cells (MSC) are important components. Indeed, the functional MSC behavior is essential to favor or impede LSC expansion and, for this reason, MSC represent a possible target for treatment of leukemias [5]. Since BM is a store of undifferentiated MSC, tumor cells precursors may affect the differentiation of MSC in the tumor niche suggesting a deep cross-talk between LSC and MSC [6]. Interestingly, despite MSC from CML patients do not express BCR–ABL [7], recent studies have reported an altered regulation of MSC in CML, showing that changes in BM microenvironmental function suppress normal hematopoietic stem cells (HSC) and provide a selective advantage to LSC[8]. Into the tumor milieu, MSC also play an important role for their immunosuppressive ability that can interfere with the immune recognition of tumor cells. Indeed, they produce and release immunoregulatory factors, including transforming growth factor β (TGF-β), prostaglandin E2 (PGE2), tumor necrosis factor α (TNFα), indolamine 2,3-dioxygenase (IDO), hemeoxygenase (HO), nitric oxidase synthase 2 (NOS2), arginase 1–2 (ARG1-2) and IL10 [5, 9–11]. MSC express programmed death ligand 1 (PD-L1) that after its engagement with PD-1 expressed on T lymphocytes leads to the inhibition of T cell activation and proliferation with an inefficient immune response [12].

Recently, we and other authors have demonstrated a significantly expanded population of myeloid derived suppressor cells (MDSC) in CML patients that is part of tumor clone and provides a favorable microenvironment in which LSC can proliferate, acquire new mutations, and evade host immuno-surveillance [13, 14]. Based on the expression of surface antigens, two main subpopulations of MDSC can be distinguished in humans: CD11b^+^ CD33^+^ CD15^+^ CD14^-^ HLA-DR^-^granulocyte-like (G-MDSC) and CD14^+^ CD15^-^ HLA-DR^-^ monocyte-like (M-MDSC) [15–17]. MDSC are able to inhibit the immune system by multiple mechanism, including production of ARG1, NOS2, reactive species of oxygen (ROS), cyclooxygenase 2 (COX2), TGFβ and immunosuppressive cytokines, such as IL6, IL10 and IL1β [18–20]. Inhibition of NK function by MDSC via down-regulation of the activating receptor NKG2D has been also reported [21]. Moreover, MDSC are able to induce regulatory T cells (T-reg) expansion [22]. The specific immunosuppressive mechanisms used by MDSC are microenvironment-dependent. We have demonstrated that in CML patients at diagnosis, G-MDSC is the most abundant subpopulation; it correlates with the percentage of T-reg and up-regulates ARG1 as mediator of the immunosuppressive action [13].

Even though the promotion of tumor growth, survival and drug-resistance induced by MSC has been widely studied, the role of MSC in MDSC expansion and activation into the BM microenvironment remains unexplored. Here, we focused our attention on CML-MSC in order to evaluate their involvement in MDSC generation into the BM microenvironment.

## Material and Methods

### Patients and sample collection

After written informed consent approved by the local ethical committee (The ethical committee of Azienda Ospedaliera Universitaria Policlinico-Vittorio Emanuele approved the current study number 34/2013/VE), samples were collected from newly diagnosed CML (n = 30) patients and age-matched healthy donors (HD; n = 20) at Division of Hematology, AOU Policlinico–OVE, University of Catania. Clinical data of CML patients included in this study are shown in Table 1.

**Table 1:** Baseline clinical characteristics of patients included in the study.

	CML (n = 30)
**Median age (range)**	58 (21–72)
**Males/Females**	18/11
**BCR/ABL transcript levels (range)**	100 (28–349)
**WBC, 10**^**2**^**/uL (range)**	89 (75–260)
**Haemoglobin, g/dL (range)**	12 (8–15)
**Platelets, 10**^**3**^**/uL (range)**	316 (107–651)
**LDH, mg/dL (range)**	1096 (345–2230)
**Liver (cm)**^**2**^ **(range)**	1.2 (0–7)
**Spleen (cm)**^**2**^ **(range)**	2.5 (0–14)
**Sokal risk group**	
*low*	14
*intermediate*	12
*high*	4

### MDSC evaluation

EDTA whole blood sample (50 μL) was stained with monoclonal antibodies (mAbs, 10 μL for each) and respective isotypic controls. The moAbs (Beckman coulter) included: CD11b FITC (clone bear-1), CD33 PE (clone D3HL60.251), CD15 PE (clone 80H5), CD14 PC5, (clone RMO52), HLA-DR- ECD (Clone Immu-357). Using sequential gating strategy, G-MDSC cells were identified as CD11b^+^CD33^+^CD15^+^CD14^-^HLA-DR^-^. The acquisition and analysis was performed with a Beckman Coulter FC-500 flow cytometer (10,000 cells were analysed).

To evaluate the suppressive ability, G-MDSC from CML patients and HD were first isolated using anti-CD66 magnetic microbeads (*MiltenyiBiotec*) and then co-cultured for three days with autologous Carboxyfluorescein succinimidyl ester (CFSE)-labeled T lymphocytes at ratio 1:4 [13, 23]. T cells were isolated by magnetic cell separation using human CD3 microbeads (Miltenyi Biotec). For T lymphocytes labeling, 5x10^5^ lymphocytes were incubated at 37°C for 20 min in 1 ml PBS containing 1 μM CFSE (BD Pharmingen). T cells were stimulated with 5 mg/mL phytohemagglutinin (PHA) and incubated for 72 hours prior to flow cytometry. Controls included a positive T cell proliferation control (Tcells plus PHA) and a negative one (T cells only). After three days, T cell proliferationwas measured by CFSE dilution andanalyzed using flow cytometry.

### MSC harvest, culture and characterization

BM mononuclear cells from HD (n = 8) and CML (n = 10) subjects were obtained after density gradient centrifugation on Ficoll and cultured in low-glucose Dulbecco’s modified Eagle’s medium supplemented with 10% heat-inactivated FBS, 100 U/ml penicillin, 100 mg/ml streptomycin and 1% L-glutamine. After 3 days in culture, non-adherent cells were removed, whereas MSCs were selected by their adherence to the plastic-ware. The cultures were maintained at 7°C and 5% CO_2_. MSCs were expanded until the third or fourth passage and then trypsinized to be used for experiments.

Selected MSC from both CML patients and HD at the third passage were also tested for MSC specific surface antigen expression. Therefore, cells were labeled using combinations of monoclonal antibodies: anti-CD34-ECD (clone 581), anti-CD90-FITC (clone F15.42.1.5), anti-CD105-PE (clone 1G2) and anti-CD45-PC5 (clone J.33). The appropriate isotopic control was also included. Labeled MSC were acquired using a Beckman Coulter FC-500 flow cytometer.

Moreover, MSC osteogenic and adipogenic ability differentiation was confirmed in two CML and HD-MSC. In brief, for osteoblastic and adipocytic differentiations, 80% confluent MSCs were grown in medium supplemented with 10mM b-glycerol phosphate (Sigma-Aldrich, St Louis, MO, USA), 50 mg/ml ascorbic acid and 10nM dexamethasone for osteoblasts, or with 10 μg/ml insulin, 0.5 mM dexamethasone, 0.5 mM isobutylmethylxanthine and 0.1 mM indomethacin for adipocytes [24, 25]. Osteocytic and adipocytic differentiation of MSC was evaluated using alkaline phosphatase and Oil-Red-O respectively (data not showed).

### MDSC induction

Human peripheral blood mononucleated cells (PBMC) were isolated from healthy volunteer donors after density gradient centrifugation on Ficoll. PBMC were cultured alone or co-cultured with MSC derived from healthy subjects and CML patients at diagnosis (1:100 ratio) [23]. MSC were seeded to achieve confluence by 7 days. After one week, PBMC were collected and G-MDSC were isolated using anti-CD66b magnetic microbeads (MiltenyiBiotec) and we defined them as“MSC-educated G-MDSC”. The phenotype of G-MDSC was confirmed by cytofluorimetric analysis. Their immunosuppressive capacity was analyzed by evaluating T cell anergy when co-cultured with autologous CFSE-labeled T cells stimulated by PHA. Controls included a positive T cell proliferation control (T cells plus PHA) and a negative one (T cells only). After three days T cell proliferation was analyzed by flow cytometry.

### Real-time RT-PCR for gene expression of MSC and MDSC

For gene expression studies, MSC were trypsinized from culture flasks both at Time 0 (cells at confluence incubated with standard medium only) and after 48 hours from start of co-culture experiments. In co-culture experiments, MSC and HD-, CML-MSC-educated G-MDSC were purified using respectively anti-CD271 and anti-CD66b magnetic microbeads (MiltenyiBiotec) [26]. After RNA extraction and reverse transcription [27], we evaluated expression of the following mRNA: ARG1, NOS2, COX2, TNFα, TGFβ, IL6, IL10 and IL1β. Their expression was assessed by TaqMan Gene Expression (Life Technologies) and quantified using a fluorescence-based real-time detection method by 7900HT Fast Start (Life Technologies). For each sample, the relative expression level of each studied mRNA was normalized using GAPDH as invariant controls.

### Statistical analysis

Statistical analysis was made with Prism Software (Graphpad Software Inc., La Jolla, CA, USA). Data were expressed as mean or SD. Statistical analysis was carried out by unpaired t-test or ANOVA test. A p-value of 0.05 was considered to indicate a statistically significant difference between experimental and control groups.

## Results

### Frequency of G-MDSC in CML patients

**Fig 1:**
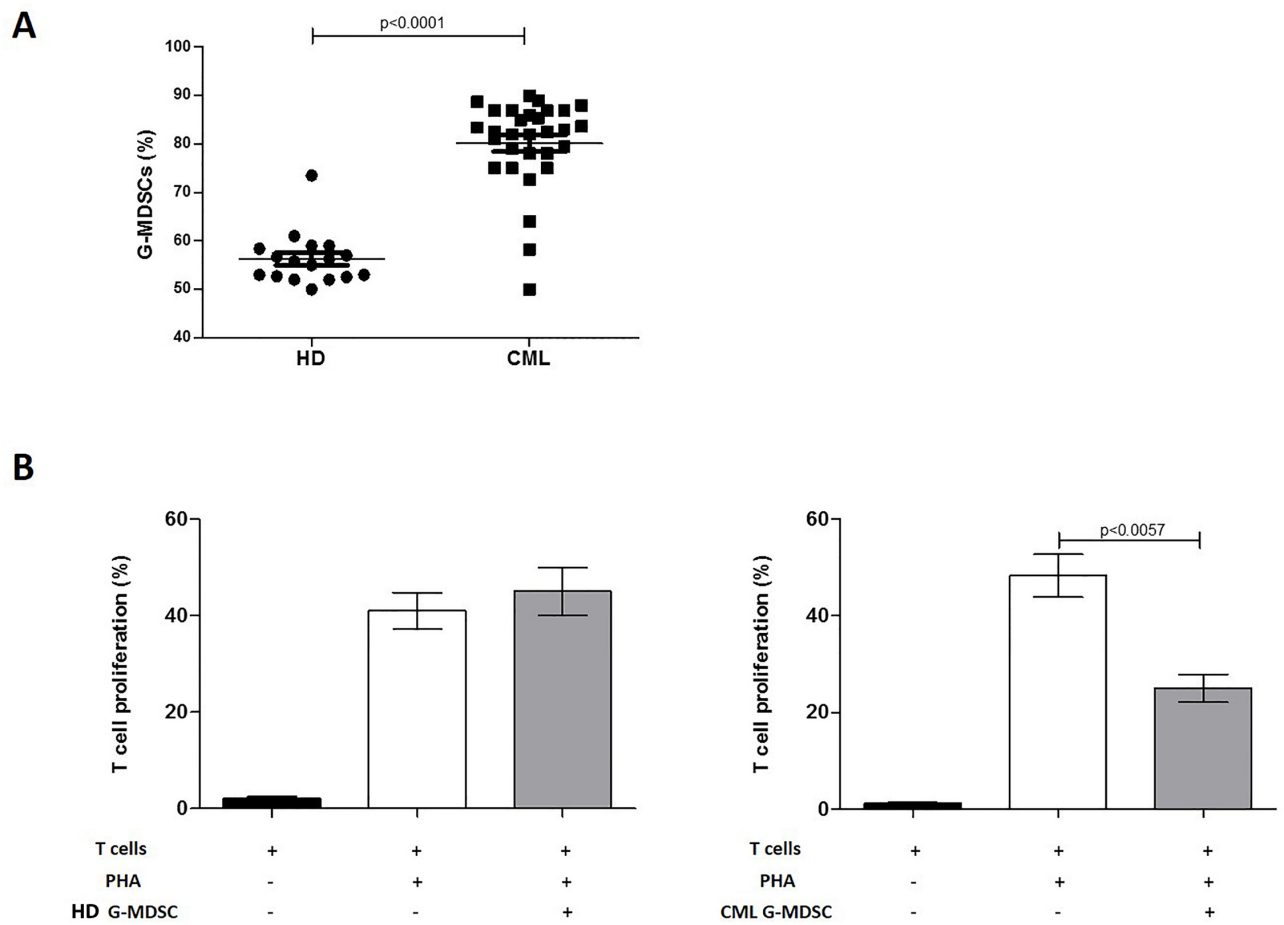
Increased frequency of G-MDSC in CML patients. **A.** Circulating G-MDSC were quantified and expressed as percentage of CD11b+CD33+CD15+CD14-HLA-DR- cells in EDTA whole blood samples using sequential gating strategy in flow cytometry. **B.** Only CML G-MDSC were able to inhibit T cell proliferation in autologous co-cultures. Mean frequency of CD3+ CFSE dim cells ± SD from four independent experiments is shown.

The percentage of G-MDSC (CD11b^+^ CD33^+^ CD15^+^ CD14^-^ HLA-DR^-^ cells) was investigated in the PB of healthy donors (HD) and CML patients at diagnosis. The frequency of G-MDSC was found to be significantly increased in CML patients compared to HD (82.5±9.6% vs 56.2±5.4%, p<0.0001) (Fig 1A). To evaluate their immunosuppressive activity, we next incubated G-MDSC with autologous CFSE+ T cells stimulated with PHA. Unlike G-MDSC isolated from HD, CML G-MDSC decreased T cells proliferation by 25±5% (p = 0.0057) (Fig 1B).

### Induction of G-MDSC by CML-MSC

**Fig 2:**
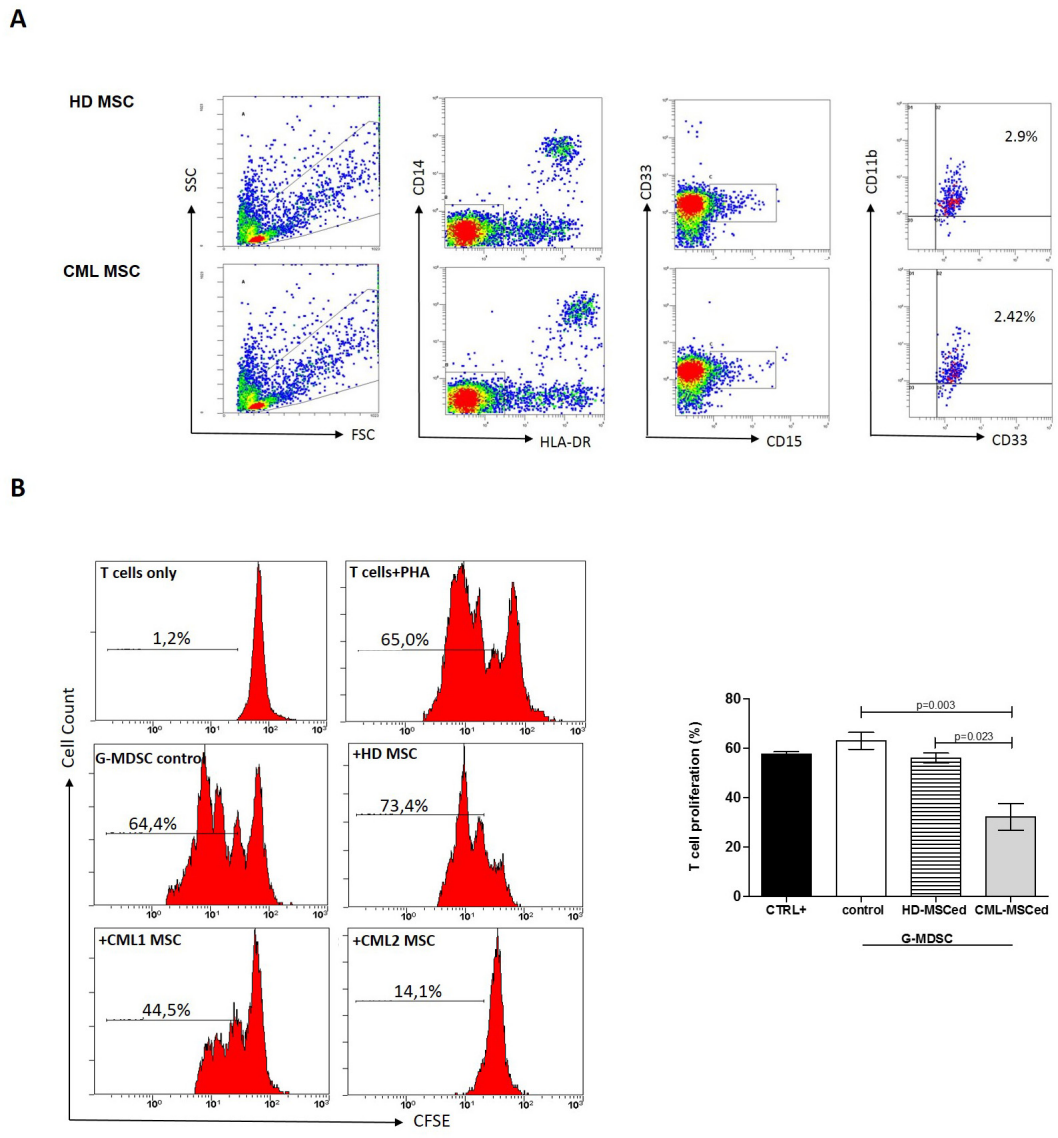
CML-MSC-educated G-MDSC are immunosuppressive. **A.** HD- and CML-MSC generate similar amount of G-MDSC. The figure shows a representative data from one experiment. Flow cytometry analysis was performed with gates set on CD11b+CD33+CD15+CD14-HLADR- cell population. **B.** MSCedG-MDSC were analyzed for their immunosuppressive activity against autologous T cells. Representative flow cytometry dot-plots show the gating strategy for each experimental condition. Only CML-MSCedG-MDSC exhibited suppressive effects compared to G-MDSC control. The data represent mean±SD of all analyzed co-cultures in triplicate.

Here, we investigated the role of MSC in expansion and/or activation of G-MDSC. Therefore, we cultured PBMC isolated from healthy subjects in medium alone or with MSC of HD or CML patients. After one week, the amount of G-MDSC was analyzed. Both HD and CML MSC accumulated similar small amount of G-MDSC (Fig 2A). Next, we analyzed immunosuppressive activity of MSC-educated G-MDSC (MSCedG-MDSC). After magnetic cell separation, the phenotype of G-MDSC was confirmed by cytofluorimetric analysis (S1 Fig). To assess their effect on T cell proliferation, MSCedG-MDSC were co-cultured with autologous CFSE+ T cells. We found that only CML-MSCedG-MDSC showed immunosuppressive ability by inhibition of T cell proliferation compared to control G-MDSC (isolated from PBMC cultured in medium alone) (32±12% vs 63±5.9%, p = 0.003). On the contrary, HD-MSCedG-MDSC did not show any suppressive effect (Fig 2B). This experiment suggests that despite neither HD- nor CML-MSC significantly expand the percentage of G-MDSC, only CML-MSC are able to generate immunosuppressive G-MDSC.

### CML-MSC up-regulate immunomodulatory factors

**Fig 3:**
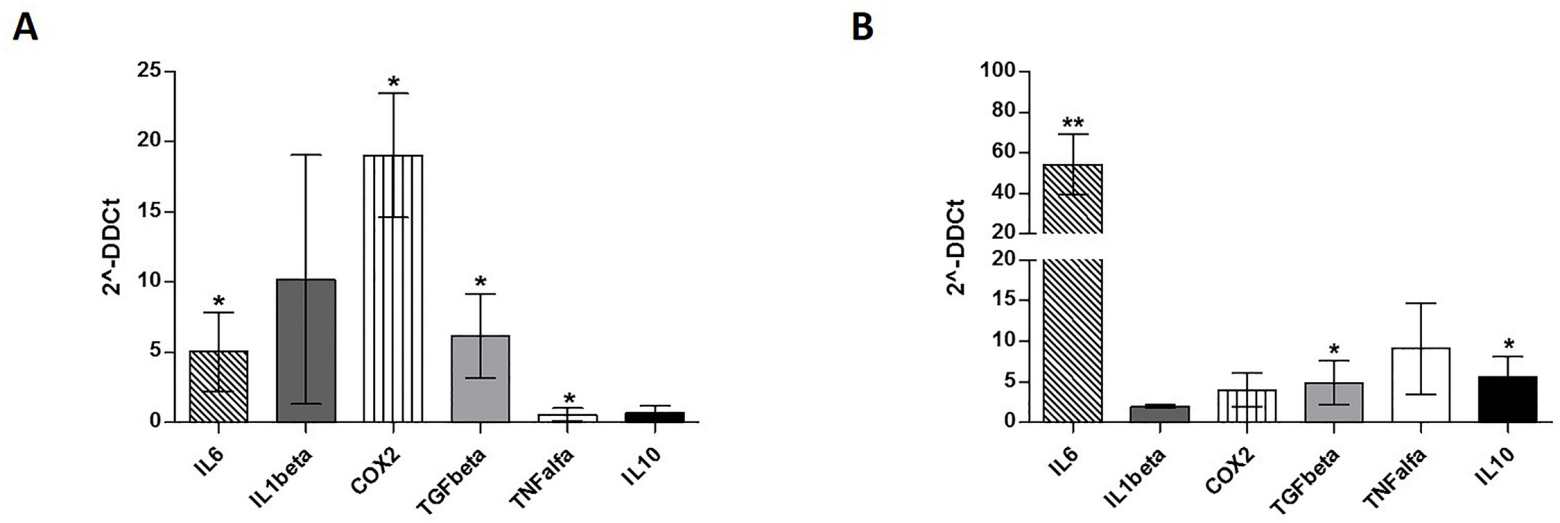
Expression of immunomodulatory factors by CML-MSC. Compared to HD-MSC, CML-MSC showedsignificant up-regulation of IL6, COX2and TGFβ at Time 0 (**A**) and overexpressed IL6, TGFβ and IL10 after 48 h of co-culture with PBMC (**B**). Calculated value of 2^-ΔΔCt in HD-MSC was 1.

It is well known that human MDSC can be induced by multiple factors present in the tumor microenvironment [28]. Immunomodulatory factors, including TNFα, TGFβ, IL6, IL10, IL1β, ARG1, NOS2 and COX2are important to reprogram immature myeloid cells to become immunosuppressive G-MDSC[29]. Therefore, we first analyzed their expression by MSC at Time 0. Despite a great variability among patients, we found a significant up-regulation of IL6 (5±2.8, p = 0.04), COX2 (19±4.4, p = 0.04) and TGFβ (6±3, p = 0.01) by CML-MSC compared to HD-ones (Fig 3A). Expression of TNFα gene was down-regulated (0.55±1, p = 0.027). After 48 h of co-culture with PBMC, CML MSCs showed up-regulation of IL6 (54.3±23, p = 0.003), TGFβ (4.8±3, p = 0.04) and IL10 (5.6±2.8, p = 0.03) expression (Fig 3B), suggesting that multiple mechanisms are involved in MDSC induction by CML MSC.

### Gene expression of immunomodulatory factors in CML-MSC-educated G-MDSC

**Fig 4:**
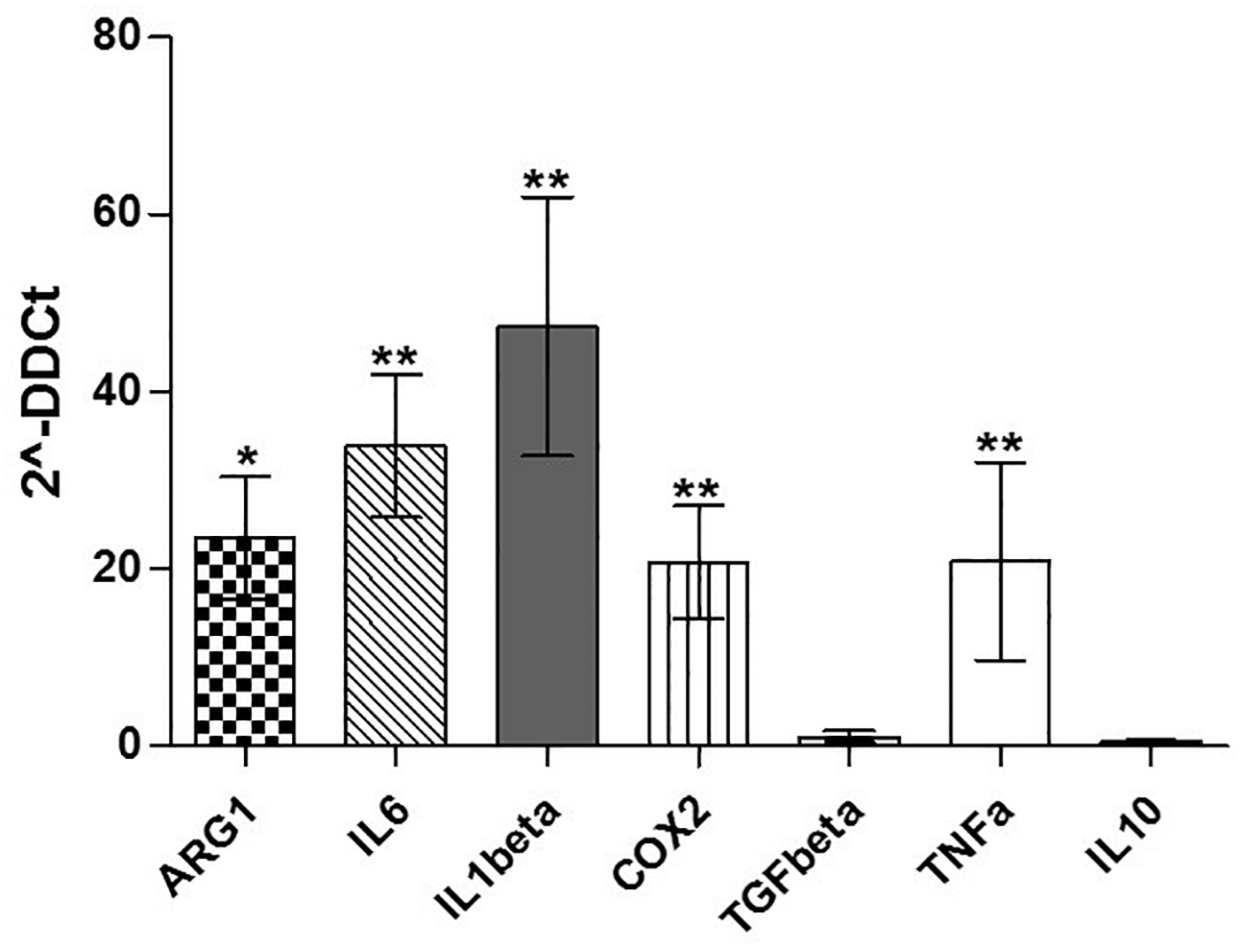
Expression of immunomodulatoryfactors by MSCedG-MDSC. Compared to HD-, CML-MSCedG-MDSC significantly upregulated ARG1, IL6, IL1β,COX2and TNFα. Calculated value of 2^-ΔΔCt in HD-MSCedG-MDSC was 1.

To test whether the changes of gene expression in CML-MSC during co-culture also occurred in CML-MSCedG-MDSC, we examined the expression of the same genes in G-MDSC that were generated from PBMC after MSC co-culture. As expected, G-MDSC activation by CML-MSC led to the up-regulation of immunomodulatory factors. CML-MSCedG-MDSC showed higher level of ARG1 (23.5±11.9, p = 0.02), IL6 (33.8±13.9, p = 0.004), IL1β (47.3±25.2, p = 0.001), COX2 (20.7±10.9, p = 0.002) and TNFα (20.8±19.3, p = 0.006) compared to HD-MSCedG-MDSC (Fig 4).

## Discussion

The ‘immune phase’ of tumor-driven inflammation involves a recruitment of antitumor effector cells to the tissue site [30]. Indeed, the accumulation of immune suppressive cells such as MDSC or T-reg in the tumor microenvironment represents a refined mechanism to evade immune response. Once established, the tumor immune suppressive microenvironment represents a consistently effective barrier to immune cell functions [31]. Some mechanisms responsible for dysfunction of immune cells are directly mediated by factors produced by tumors, whereas others result from tumor-associated microenvironment, including IL-1β, IL-4, IL-6, IL-10, IFN-γ and TGF-β. These molecules re-program immature myeloid cells to become immunosuppressive [29, 32, 33].

Analyzing CML patients at diagnosis, we have demonstrated that the frequency of G-MDSC in PB was higher compared with healthy subjects and these cells showed immunosuppressive ability. On the contrary, G-MDSC isolated from HD were not able to inhibit T lymphocyte proliferation.

**Fig 5:**
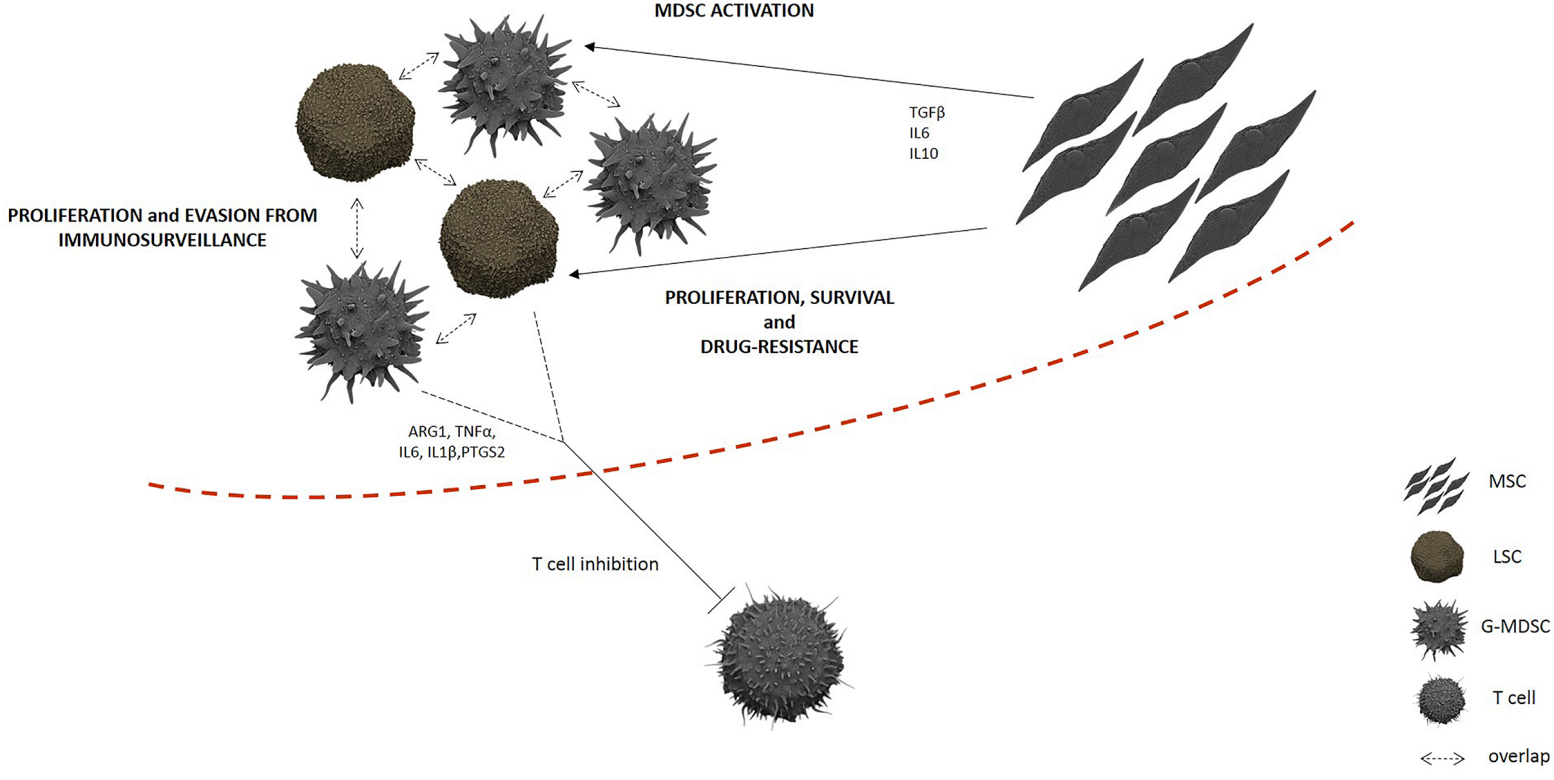
Immunosuppressive niche in CML BM. Even though the expansion of G-MDSC is, at least in part, linked to tumor proliferation in CML patients because of their overlap with leukemic clone, CML-MSC play a centralrole in G-MDSC activation, contributing to create an effective barrier against immune cells.

The contribution of MSC to cancer hallmarks is well documented [34]. Indeed, among other abilities, MSC show a diversity of immune modulatory actions [35]. Our present experiments indicate that MSC contribute to transform the BM microenvironment into an immune suppressive one by orchestrating MDSC. Indeed, despite HD- and CML-MSC generate similar amount of G-MDSC, the ability to suppress T lymphocyte proliferation was found only for G-MDSC that were generated after a co-culture with MSC derived from CML patients. No suppressive effect was ever observed incubating T lymphocytes with HD-MSCedG-MDSC, demonstrating that CML-MSC are functionally different from HD-MSC. Sánchez et colleagues showed that immunosuppressive properties of MSC evolve along neoplastic transformation [36]. Using a murine model, the authors observed that both normal and in vitro transformed MSC accumulated similar percentage of G-MDSC, but murine MDSC (IL4Rα^high^/GR1^low^) differentiated in presence of transformed MSC, exhibited an enhanced inhibitory effect on T cell proliferation. In human, it is still an open question to define a different role of tumor versus healthy MSC. When compared with their normal counterpart, CML-MSC show normal morphology, phenotype and karyotype but appear impaired in immunomodulatory function. Indeed, previous studies have demonstrated that MSC from CML patients showed very limited inhibitory effects but they might be a cause for an abnormal hematopoietic environment [37]. For the first time, our data provide evidence that unlike MSC derived from healthy subjects, CML-MSC are able to generate G-MDSC, demonstrating an evolving concept regarding the contribution of MSC in the CML immune surveillance evasion (Fig 5).

Exploring the immunomodulatory factors expressed by CML-MSC at Time 0, we found a significant up-regulation of COX2, TGFβ, and IL6 compared to HD-MSC, with a great variability among patients. These results reveal an acquired impairment by CML MSC in their immunomodulatory functions. In addition, during co-culture with PBMC, CML-MSC significantly up-regulated TGFβ, IL6, and IL10 expression, that are among the cytokines described to induce MDSC expansion [29, 38, 39].

G-MDSC inhibit immune system by multiple mechanisms, mostly through inhibition of T cell activation and expansion, and the specific mechanisms used are microenvironment-dependent [40]. To investigate the influence of MSC on the expression of the immunomodulatory genes in MSCedG-MDSC, we then examined their expression in G-MDSC before incubation with T lymphocytes. Compared to HD-, CML-MSCedG-MDSC up-regulated expression of ARG1, TNFα, IL1β, COX2 and IL6, providing thus evidence that CML-MSC transform myeloid cells in immunosuppressive ones. Indeed, up-regulation of ARG1 is one of the main mechanisms of MDSC-induced immunosuppression [41] and this protein is highly expressed by both MDSC and polymorphonuclear leukocytes in CML patients [13, 14]. TNFα has been shown to arrest differentiation of immature myeloid cells and increase MDSC suppressive activity [42]. Also up-regulation of COX2 has been reported as mechanism of MDSC-mediated immunosuppression [19]. In addition, more recently, IL-6 has been found to stimulate NF-κB-mediated IDO upregulation in MDSC [43].

Collectively, our findings show how CML-MSC directly orchestrate immune escape by driving MDSC activation in the tumor microenvironment. Whether the alteration of the immunoregulatory abilities of MSC reveals an acquired capacity by MSC themselves, as consequence of a neoplastic transformation, or derived by interaction with tumor cells is a relevant question for clinical oncology. Since CML MSC do not express BCR/ABL and here we demonstrate that CML MSC obtained after in vitro expansion are able to induce G-MDSC generation, we conclude that CML MSC certainly have a constitutive functional alteration.

Despite the introduction of tyrosine kinase inhibitor drugs, that have dramatically improved the prognosis of CML patients, many problems remain to be solved because of the inability of these drugs to eradicate the leukemia stem cell (LSC) compartment [44]. Zhanget and colleagues demonstrated the contribution of leukemia-induced alterations in the BM microenvironment that suppress normal HSC and provide a selective advantage to LSC [8]. Although TKI treatment reduces normal HSC inhibition by leukemic cells and facilitates their regrowth, it does not completely reverse leukemia-associated changes in the microenvironment [8]. Therefore, it is important to determine the mechanisms underlying these persistent changes and how leukemia-related alterations affect LSC response to TKI. It seems reasonable to hypothesize that interactions of the tumor with MSC are a critical factor for tumor promotion. By inducing G-MDSC activation, CML-MSC are relevant in regulating T lymphocytes-mediated leukemia surveillance, becoming a potential target to treat and act on leukemia microenvironment.

## Supporting Information

S1 Fig Purity of educated G-MDSC after magnetic cell separation. After separation, the cells were incubated with fluorescently labeled anti-CD11b, anti-CD15, anti-CD33, anti-CD14 and anti-HLADR antibodies, and the purity of the cells was analyzed by flow cytometry. The figure reports the representative flow cytometry dot plots showing the purity of educated G-MDSC (87,3%).
(PDF)
