# Supplementary figures and images for: Mesenchymal Stem Cells (MSC) Regulate Activation of Granulocyte-Like Myeloid Derived Suppressor Cells (G-MDSC) in Chronic Myeloid Leukemia Patients
Source: PLoS One. 2016 Jul 8;11(7):e0158392. doi: 10.1371/journal.pone.0158392 (PMC4938578; doi:10.1371/journal.pone.0158392)

SS

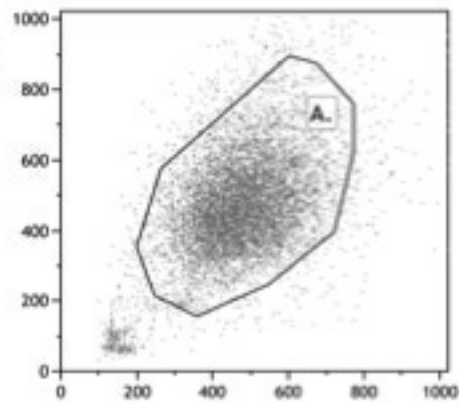

FS

CD15

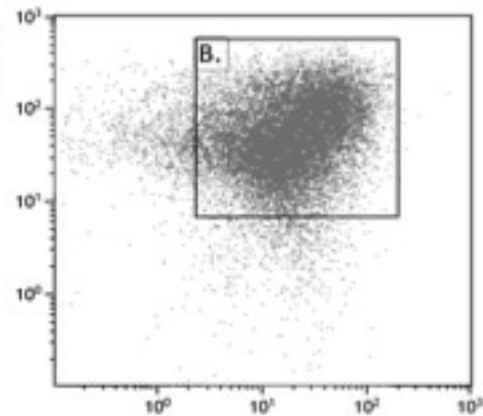

CD11b

CD33

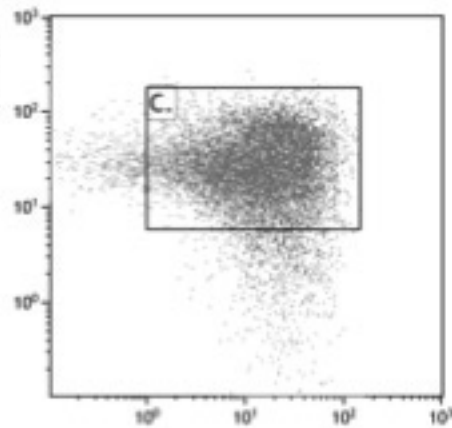

CD11b

CD14

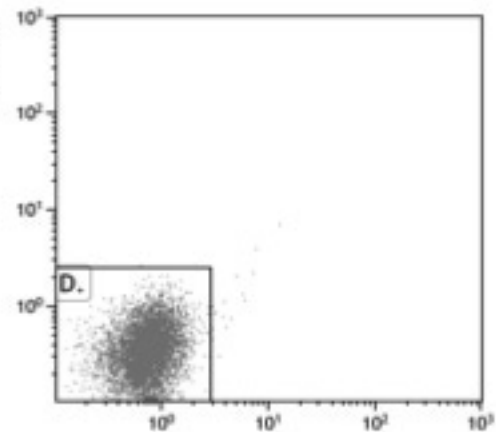

HLA-DR

Supplement: S1 Fig — After separation, the cells were incubated with fluorescently labeled anti-CD11b, anti-CD15, anti-CD33, anti-CD14 and anti-HLADR antibodies, and the purity of the cells was analyzed by flow cytometry. The figure reports the representative flow cytometry dot plots showing the purity of educated G-MDSC (87,3%). (PDF) [file pone.0158392.s001.pdf]
